# GDF10 exacerbates metastatic burden and cachexia in murine models of cancer

**DOI:** 10.3389/fphys.2026.1773275

**Published:** 2026-04-24

**Authors:** Lauren S. James, Alastair A. E. Saunders, Chris Karagiannis, Hongwei Qian, Robin L. Anderson, Rachel E. Thomson, Craig A. Goodman, Paul Gregorevic

**Affiliations:** 1 Centre for Muscle Research, and Department of Anatomy and Physiology, The University of Melbourne, Parkville, VIC, Australia; 2 Metastasis Research Laboratory, Olivia Newton-John Cancer Research Institute, Heidelberg, VIC, Australia; 3 School of Cancer Medicine, La Trobe University, Bundoora, VIC, Australia; 4 Department of Clinical Pathology, The University of Melbourne, Parkville, VIC, Australia; 5 Department of Neurology, The University of Washington School of Medicine, Seattle, WA, United States

**Keywords:** adeno-associated virus, BMP3b, cachexia, cancer, GDF10, metastasis, skeletal muscle

## Abstract

Metastasis and cancer-induced cachexia significantly reduce survivorship and quality of life for cancer patients. GDF10 (BMP3b) is a TGF-ß superfamily ligand with little knowledge of its role in cancer progression. Some studies have shown that GDF10 exerts tumor-suppressive effects in a range of cancer types and also plays a protective role against muscle wasting. Basal transcription of GDF10 was described previously to be downregulated in both primary tumors and cachectic muscle. Here, we set out to investigate the therapeutic potential of GDF10 in the 4T1.2 mouse model of breast cancer metastasis and in the C-26 mouse model of cancer cachexia, hypothesizing that GDF10 would ameliorate both metastatic and cachectic disease pathology. Systemic rAAV6:GDF10 administration to mice did not alter primary tumor growth; however, metastatic burden was increased in the mice bearing 4T1.2 tumors. Similarly, increased intramuscular rAAV6:GDF10 expression exacerbated skeletal muscle wasting in C-26 tumor-bearing mice. These results contradicted our initial hypothesis and highlight the complexity of signaling mechanisms utilized by BMP family ligands. Our data point to the need for more research to understand how to target GDF10 in anti-cancer therapy.

## 1 Introduction

Most cancer-related deaths are caused by metastatic tumors that develop in organs distal to the primary tumor site (Lambert et al., 2017). Standard cancer treatments are often successful in treating primary cancers, but for metastatic disease, existing treatments may no longer be effective. The limited efficacy of conventional chemotherapy and radiotherapy in treating metastatic cancer underscores the need to elucidate the biological mechanisms underlying metastasis, to help inform the development of more effective therapeutic strategies. Cachexia is another aspect of cancer pathology that impacts the quality of life for cancer patients (Boire et al., 2024). Cachexia is a multifactorial and multi-organ syndrome that is characterized by the loss of functional skeletal muscle and adipose tissue, resulting in weakness and fatigue, reduced tolerance to aggressive treatment strategies and accelerated death in cancer patients (Baracos et al., 2018).

Signaling via the Transforming Growth Factor-β (TGF-ß) network has been investigated in multiple disease pathologies (Xie et al., 2018; Tzavlaki and Moustakas, 2020), including in cancer. It is reported to either promote or suppress cancer cell proliferation (Bashir et al., 2015; Eckhardt et al., 2020b), the progression of metastatic disease (Padua et al., 2008; Drabsch and Ten Dijke, 2011), as well as the regulation of muscle function (Xie et al., 2018; Tzavlaki and Moustakas, 2020). The TGF-ß superfamily contains over 30 secreted extra-cellular ligands that bind cell surface receptors, leading to the phosphorylation of Smad transcription factors (Shi and Massague, 2003). The subsequent cellular response is dependent on the specific ligand/receptor combination. For example, ligands such as activins and inhibins lead to increased phosphorylation of Smads 2 and 3, which are associated with the promotion of metastasis (Padua et al., 2008; Drabsch and Ten Dijke, 2011) and muscle atrophy (Chen et al., 2017). Conversely, bone morphogenetic proteins (BMPs) predominantly signal through Smads 1, 5, and 9 downstream of BMPRII and ALK1/2/3/6 receptors resulting in metastatic suppression and muscle hypertrophy (Derynck and Zhang, 2003; Chi et al., 2019; Winbanks et al., 2013; Sartori et al., 2013; Goodman and Hornberger, 2014). However, due to the dichotomous nature of BMP ligands in certain cancer contexts, BMP activity has also been associated with enhanced metastatic potential (Yang et al., 2005; David and Massague, 2018). Thus, it is important to define the actions of different BMP family members.

GDF10, also referred to as BMP3b, is an example of a bone morphogenetic protein family member whose signaling pathway and function in cancer has not yet been fully deciphered. Previous reports have shown that the expression of GDF10 was reduced in malignant tumors compared to normal tissue (Dai et al., 2004; Cheng et al., 2016). Furthermore, *in vivo* studies have characterized GDF10 as a tumor suppressor, shown to reduce primary tumor growth in nasopharyngeal carcinoma (He et al., 2022). Although GDF10 has been examined in primary cancers, its role in metastatic disease has not been explored in an *in vivo* setting. Therefore, the first aim of this study was to determine the effect of increased GDF10 expression in an *in vivo* model of metastatic breast cancer. Other studies have reported that transgenic overexpression of GDF10 in mice or administration of recombinant GDF10 is able to reduce muscle atrophy and improve muscle function in sarcopenia (Kurosawa et al., 2021; Uezumi et al., 2021). Thus, the second aim of this project was to assess whether increased GDF10 expression could mitigate the muscle wasting associated with cancer cachexia. Since both metastasis and muscle wasting have been linked to increased Smad2 signaling and/or reduced Smad1/5/9 signaling (Chen et al., 2014; Sartori et al., 2021), we hypothesized that GDF10 would suppress metastatic and cachectic progression by inhibiting Smad2 activity and enhancing Smad1/5/9 signaling.

## 2 Methods

### 2.1 Reagents

All reagents were purchased from Merck Life Science Pty Ltd (Victoria, AUS) unless otherwise stated. Recombinant adeno-associated viral vectors (rAAV) were generated as described previously (Hagg et al., 2020).

### 2.2 Ethics Statement

All experimental protocols were approved by the Animal Ethics Committee of the University of Melbourne and conducted in accordance with the Australian Code of Practice for Care and Use of Animals for Scientific Purposes as maintained by the National Health and Medical Research Council (Australia).

### 2.3 Cell lines and tissue culture

The 4T1.2 bone metastatic breast tumor cell line was derived from the syngeneic Balb/c mouse mammary 4T1 cell line (Lelekakis et al., 1999). The 4T1.2 cell line was transfected with a stable lentivirus that expresses mCherry (pLV-mCherry) for subsequent detection in tissues. The Colon 26 (C-26) carcinoma cells were derived from Balb/c mice, and tumor pieces were utilized as previously described (Aulino et al., 2010; Winbanks et al., 2016; Sartori et al., 2021).

### 2.4 Animals

Experiments were conducted in 8-week-old Balb/c mice sourced from the Walter and Eliza Hall Institute of Medical Research (Victoria, Australia) or Australian BioResources (ABR, New South Wales, Australia). Surgical procedures were performed under inhalation anesthesia with isoflurane in medical oxygen, and post-operative meloxicam analgesia. Mice were housed in the Biomedical Science Animal Facility at The University of Melbourne under a 12-h light/dark cycle with standard laboratory chow with water available *ad libitum.* Mice of the same sex were randomly assigned to experimental groups.

### 2.5 Assessment of metastatic burden

Orthotopic mammary tumor growth was achieved through injection of 1x10^5^ mCherry-expressing 4T1.2 cells (Lelekakis et al., 1999) suspended in 15µl Hanks Buffered Salt Solution (HBSS; Thermo Fisher Scientific) into the 4^th^ inguinal fat pad of female Balb/c mice. Mice received an intravenous tail vein injection of rAAV vectors two weeks prior to injection of tumor cells. rAAV: GDF10 and a non-coding control rAAV (Con) were prepared in 120µl of HBSS at a concentration of 1x10^13^ vector genomes (vg) per ml. Primary tumors were resected at 300-400mm^3^, as measured using electronic calipers. Mice were humanely euthanized at set timepoints for metastatic burden analysis (as described in figure legends), or earlier if they developed signs of ill-health due to metastatic disease. Assessment of metastatic burden by quantitative PCR was performed as previously described (Saunders et al., 2025).

### 2.6 Assessment of cachexia progression

C-26 carcinoma tissue implantation was achieved by inserting 1mm^3^ C-26 tissue pieces into the dorsal right flank of male Balb/c mice. At the same time, mice were administered rAAV: GDF10 via intra-muscular injection of the tibialis anterior (TA) muscle at a dose of 5x10^9^ vector genomes diluted in 30μl of PBS. A control vector (rAAV: Con) was injected into the contralateral leg. Mice were subsequently monitored for progressive weight loss and were euthanized by cervical dislocation while under anesthesia 28 days after tumor implantation. TA muscles were collected for further analysis.

### 2.7 qRT-PCR

RNA or genomic DNA (gDNA) was extracted from frozen TA or lung samples respectively, as described previously (Hagg et al., 2020; Chi et al., 2024b; Saunders et al., 2025). The Bio-Rad CFX384 PCR system (Bio-Rad Laboratories) was used to perform qPCR as described previously (Sartori et al., 2021; Saunders et al., 2025). The following gene sequences were used and obtained through Invitrogen, Sigma Merck or IDT:

*mGAPDH*: F; CCTTCTCCATGGTGGTGAAGAC, R; CACCATCTTCCAGGAGCGAG.
*mGDF10*: F; TGAGAAGTCACAACCGAAGA, R; GAGGATCATTTCTGAGTCTTG.
*mCherry:* F; GACCACCTACAAGGCCAAGAAG, R; AGGTGATGTCCAACTTGATGTTGA,
Probe;/56FAM/CAGCTGCCC/ZEN/GGCGCCTACA/3IABkFQ/.
*mVimentin:* F; AGCTGCTAACTACCAGGACACTATTG, R; CGAAGGTGACGAGCCATCTC, Probe;/5YakYeI/CCTTCATGT/ZEN/TTTGGATCTCATCCTGCAGG/3IABkFQ/.
*hGDF10:* F; TAAGATCGTTCGTCCATCC, R; CACATTCCGATTCTCATCC.

### 2.8 Immunoblot analysis

Primary tumor and TA muscles were homogenized, and protein extracted as described previously (Sartori et al., 2021). Homogenates were run on pre-cast 4-15% Bis-Tris gels (Bio-Rad, New South Wales, Australia) at a constant voltage of 150V and transferred onto polyvinylidene difluoride membranes (Millipore). The immunoblotting and transfer proceeded as described previously (Saunders et al., 2025). The following antibodies were used and obtained through the following companies: (pSmad1/5/9; Rabbit; 1:1000; Cell Signaling; #9516. pSmad2; Rabbit; 1:1000; Cell Signaling; #3108. pSmad3; Rabbit; 1:2000; Abcam; #ab63403. hGDF10; Goat; 1:1000; R&D Systems; #AF1453. K48; Rabbit; 1:1000; Cell Signaling; #4289. Smad1; Rabbit; 1:1000, Cell Signaling, #9743. Smad2/3; Rabbit; 1:1000; Cell Signaling; #8685).

### 2.9 Hematoxylin and Eosin staining

Hematoxylin and Eosin (H&E) staining was used to determine the morphological characteristics of skeletal muscle. Cryopreserved TA muscle sections (10µm) were fixed, stained and analyzed as described previously (Hagg et al., 2020).

### 2.10 Immunofluorescence and microscopy

Transverse cryosections of TA muscles (10 µm thickness) were fixed in ice cold 100% methanol and air dried. Slides were blocked in PBS containing 0.05% Tween-20 and 5% normal goat serum (NGS) in a humidity chamber. Sections were then treated with primary antibodies (anti-laminin; Rabbit; 1:200; Sigma Merck; #L9393) for 90 min at room temperature in a humidity chamber. After incubation, slides were washed for 5 min in PBS containing 0.05% Tween-20 and twice in PBS for 5 min each. Slides were then incubated in secondary antibodies for 90 min at room temperature in a humidity chamber, followed by washing for 5 min in PBS containing 0.05% Tween-20 and then twice in PBS for 5 min each. Slides were mounted with Mowiol 4–88 mounting medium and imaged on an Axio imager 2 (Zeiss). Cross sectional area analysis and minimum Feret’s (min. Feret’s) diameter were analyzed and measured as previously described (Saunders et al., 2025).

### 2.11 Statistical analysis

To compare between treatments and mice, unpaired t-tests were performed. To compare two different treatments over time, a two-way ANOVA was performed with a Sidak’s *post-hoc* test. To compare between multiple timepoints, a one-way ANOVA with Dunnett’s *post-hoc* test was performed. All statistical analyses were performed using GraphPad Prism v.10 (GraphPad, California, USA). Asterisk symbols (*) were used to indicate significant differences unless otherwise stated. P<0.05 was considered statistically significant and differences were denoted on graphs by *p<0.05, **p<0.01, ***p<0.001, ****p<0.0001. Data are presented as mean ± SEM.

## 3 Results

### 3.1 Effects of GDF10 on primary tumor growth and secondary metastatic burden in a model of triple-negative breast cancer

**Figure 1:**
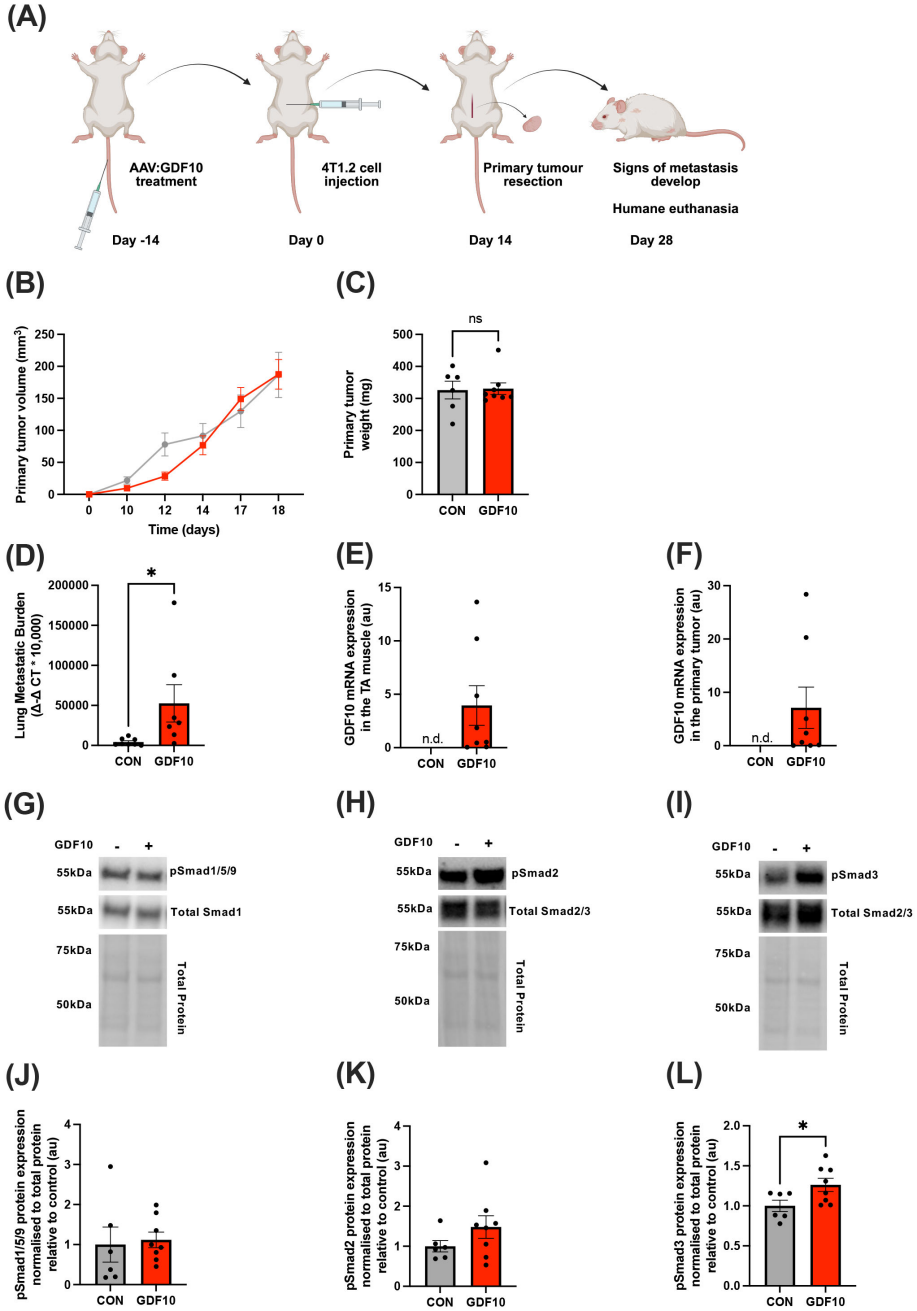
Intravenous delivery of rAAV: GDF10 does not impact primary tumor growth but increases metastasis in 4T1.2 tumor-bearing mice. **(A)** Graphical figure showing experimental outline. **(B)** Primary tumor growth until tumor resection surgery. **(C)** Primary tumor weights at resection **(D)** mCherry gDNA expression in whole lungs **(E)** GDF10 mRNA expression in tibialis anterior muscles. **(F)** GDF10 mRNA expression in primary tumors. **(G)** Western blot analysis of 4T1.2 mouse primary tumors to determine differences in the phosphorylation of Smad1/5/9 **(G, J)**, Smad2 **(H, K)** and Smad3 **(I, L)**, relative to control muscles. Unpaired t-tests were used to determine differences between GDF10-treated and untreated groups. Statistically significant differences were denoted by *p<0.05, **p<0.01, ***p<0.001, ****p<0.0001. Data presented as mean ± SEM.

Eight-week-old female Balb/c were pre-treated with a single administration of rAAV: GDF10 via tail-vein injection 14 days prior to orthotopic injection of 4T1.2mCherry cells (**Figure 1A**). Tumors were resected 14 days later when approximately 300-400mm^3^. Mice were humanely euthanized a further 14 days later, and metastatic tumor burden in the lung was assessed by gDNA extraction and qPCR. There was no significant difference in primary tumor growth rate (Day 18: p>0.9999, **Figure 1B**) or in primary tumor weight at resection in GDF10-treated mice compared to control mice (p=0.8932, **Figure 1C**). However, GDF10 treatment significantly increased lung metastatic burden compared to the control mice (p=0.0445, unpaired t-test, **Figure 1D**). To confirm systemic rAAV: GDF10 expression in the 4T1.2 treated group, GDF10 mRNA was quantified in the tibialis anterior muscle and in the primary tumor (**Figures 1E, F**). Western blot analysis of the primary tumors showed no significant change in the abundance of phosphorylated Smad1/5/9 proteins (p=0.7933, **Figures 1G, J**) or phosphorylated Smad2 protein (p=0.1936, **Figures 1H, K**). However, there was a significant elevation in Smad3 phosphorylation within the primary tumor relative to the control mice (p=0.0404, unpaired t-test; **Figures 1I, L**).

### 3.2 Effects of GDF10 on cachexia in a model of implanted colorectal carcinoma

**Figure 2:**
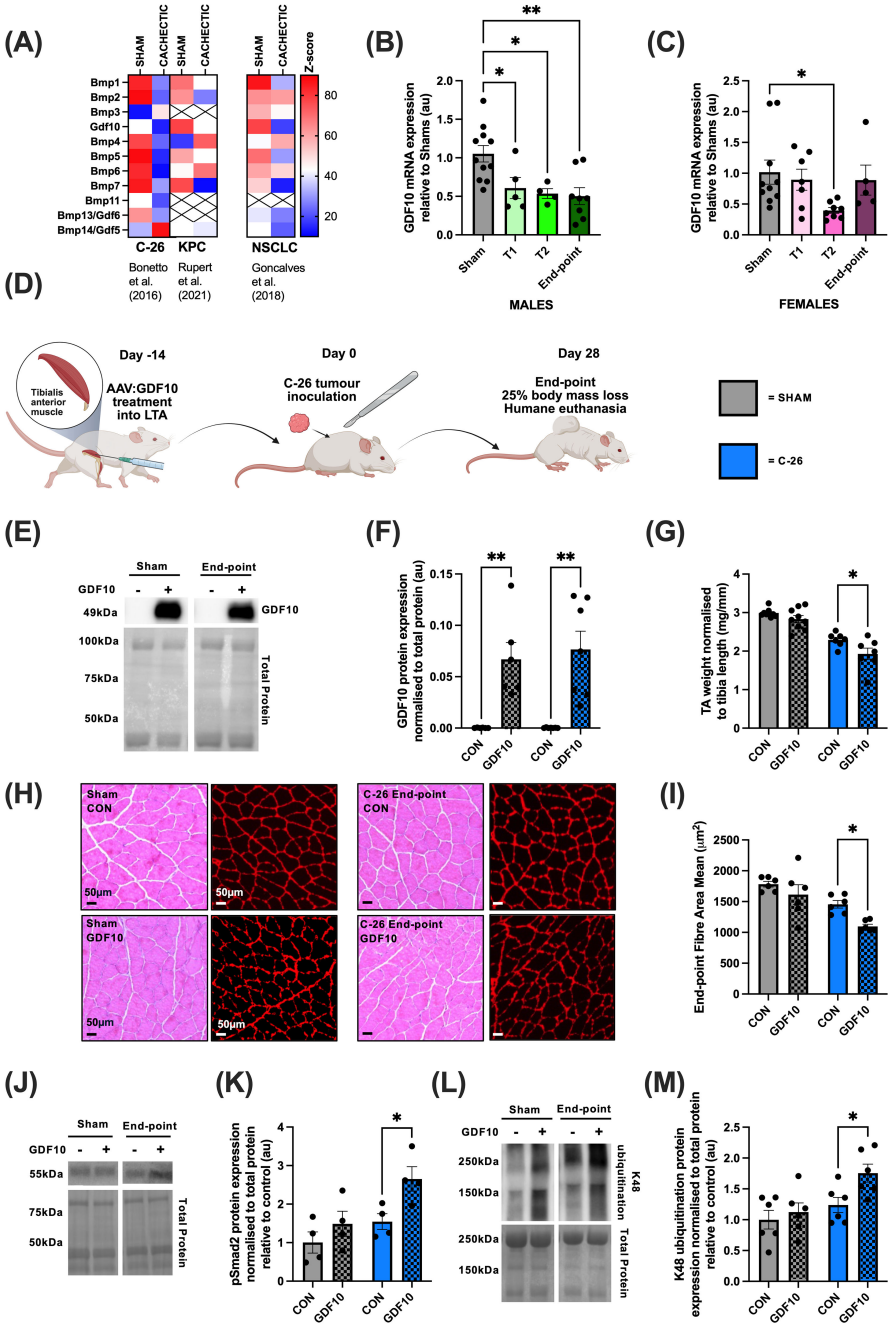
Intramuscular delivery of rAAV: GDF10 exacerbates muscle mass loss in a C-26 tumor-bearing mouse model of cachexia. **(A)** Supplementary dataset screen of BMP ligands in tumor-bearing cachectic muscle compared to control. **(B)** mGDF10 mRNA expression in tibialis anterior muscles was assessed by qRT-PCR from male C-26 tumor-bearing mice and non-tumor bearing shams. **(C)** mGDF10 mRNA expression in tibialis anterior muscles was assessed by qRT-PCR from female C-26 tumor-bearing mice and non-tumor bearing shams. **(D)** Graphical figure detailing pre-treatment, C-26 tumor inoculation and end-point at 25% body mass loss. **(E)** Western blot analysis of sham and C-26 tibialis anterior muscles to determine protein levels of GDF10. **(F)** Abundance of GDF10 protein and total protein was analyzed and normalised to control. **(G)** GDF10-treated and control tibialis anterior muscle weights from sham and C-26 mice normalised to tibial length. **(H)** Representative images of hematoxylin and eosin (H&E) stained TA cross-sections from sham and C-26 mice. **(I)** Mean fiber area of sham and C-26 mice. **(J)** Western blot analysis of sham and C-26 tibialis anterior muscles to determine Smad2 phosphorylation. **(K)** The abundance of phosphorylated Smad2 relative to total protein in sham and C-26 mice. **(L)** Western blot analysis of sham and C-26 tibialis anterior muscles to determine differences in K48-linked ubiquitination against total protein. **(M)** K48-linked ubiquitination and total protein was quantified to determine differences between sham and C-26 mice. One-way ANOVA with Dunnett’s *post hoc* test **(B, C)**, and two-way ANOVA with uncorrected Sidak’s *post hoc* test **(F, G, I–M)** were used to determine differences between sham and C-26 groups and control-treated and GDF10-treated groups. Statistically significant differences were denoted by *p<0.05, **p<0.01, ***p<0.001, ****p<0.0001. Data presented as mean ± SEM.

To examine the regulation of GDF10 expression in a cachectic muscle environment, published transcriptomic datasets accompanying the mouse cancer cachexia studies of Goncalves et al. (2018); non-small cell lung cancer, NSCLC) (Goncalves et al., 2018), *Bonetto et al.* (2016; Colon-26 Carcinoma, C-26) (Bonetto et al., 2016) and Rupert et al. (2021); pancreatic adenocarcinoma, PDAC) (Rupert et al., 2021) were analyzed for changes in the expression GDF10. As shown in **Figure 2A**, GDF10 expression was lower in cachectic muscle compared to the muscles of non-cachectic controls in all three different types of cancer, indicating a conserved response of GDF10 expression with cancer cachexia.

To build on these results, we examined the time course of GDF10 expression during the progression of C-26 tumor-induced cachexia in 8-week-old male and female Balb/c mice implanted with C-26 tumor pieces into the dorsal right flank. Mice were then monitored for progressive weight loss over a 4-week period. Muscles were collected at pre-determined time points; where time-point 1 (T1) reflected initial tumor palpation, time-point 2 (T2) reflected 10% body mass loss, and endpoint reflected 25% body mass loss and humane euthanasia of the mice. By qRT-PCR analysis, mRNA expression of mGDF10 was found to be significantly downregulated across all three time-points of cachexia in male mice compared to shams (T1: p=0.0395, T2: p=0.0261, Endpoint: p=0.0026, Dunnett’s *post hoc* test, **Figure 2B**), whereas in female cachectic mice, mGDF10 mRNA expression was only significantly decreased at T2 compared to sham (p=0.0309, Dunnett’s *post hoc* test, **Figure 2C**). These experimental findings reflect observed analyses from Goncalves et al. (2018) *(*Goncalves et al., 2018*)*, Bonetto et al. (2016) (Bonetto et al., 2016), and Rupert et al. (2021) *(*Rupert et al., 2021*)*, indicating that reduced GDF10 mRNA in muscle is associated with cachectic pathology.

As GDF10 expression was decreased in C-26 tumor-bearing male mice across all time-points including end-point, the therapeutic potential of GDF10 was assessed by pre-treating male mice with a single intramuscular injection of rAAV: GDF10 into one tibialis anterior muscle and rAAV: Con into the contra-lateral muscle. This pre-treatment approach was used to establish an elevated abundance of GDF10 in the muscle prior to tumor inoculation. Fourteen days later, mice were implanted with a C-26 tumor piece into the dorsal right flank and were humanely euthanized 28 days post-tumor inoculation (**Figure 2D**). Western blot analysis demonstrated that there was a significant increase in muscle GDF10 protein abundance in the treated limbs of sham and end-point tumor-bearing mice compared to control muscles (Sham: p=0.0068, C26: p=0.0015, Sidak’s *post-hoc* test, **Figures 2E, F**). However, contrary to the hypothesis, increased GDF10 expression exacerbated the cachexia-induced muscle mass loss in mice bearing C-26 tumors compared to the contralateral control leg (main C26 effect: p<0.0001 main GDF10 effect: p=0.0034, C26: control vs GDF10: p=0.0111, Sidak’s *post-hoc* test, **Figure 2G**). Importantly, histological analysis of the GDF10-treated and control muscles revealed no visible signs of immune infiltrate or pathology, but consistent with the decrease in muscle mass, quantification of laminin label on cryosections (**Figure 2H**) revealed a significant decrease in mean muscle fiber cross-sectional area in treated legs compared to untreated legs in C-26 mice (main C26 effect: p=0.0012, main GDF10 effect: p=0.0109, C26: control vs GDF10: p=0.0264, Sidak’s *post-hoc* test, **Figure 2I**).

Given the finding that increased GDF10 abundance exacerbated muscle atrophy during late-stage cachexia associated with C-26 carcinoma implantation, western blot analysis was used to reveal potential mechanisms. Smad2 phosphorylation was significantly increased in GDF10-treated tibialis anterior muscles compared to control muscles in C-26 end-point mice (main C26 effect: p=0.0478, main GDF10 effect: p=0.0098, C26: control vs GDF10: p=0.0209, Sidak’s *post-hoc* test, **Figures 2J, K**). Global changes to protein lysine K48-linked polyubiquitination was used as a surrogate marker of potential changes to the rate of protein degradation, with K48-linked ubiquitination significantly increasing in GDF10-treated muscles compared to the contralateral control muscles in C-26 end-point mice (main C26 effect: p=0.0235, main GDF10 effect: p=0.0228, C26: control vs GDF10: p=0.0239, Sidak’s *post-hoc* test, **Figures 2L, M**). These data indicate that increased GDF10 may exacerbate cancer-induced muscle atrophy, in part, by increasing protein degradation, which may be linked to increased Smad2-mediated signaling.

## 4 Discussion

The role of GDF10 in cancer progression is not fully understood. Therefore, we aimed to investigate the effect of elevated GDF10 expression in metastasis and cancer-induced cachexia. Previous reports have suggested that GDF10 acts via canonical BMP/Smad1/5/9 signaling (Hino et al., 2004). In some contexts, BMP-Smad1/5/9 signaling is known to exert an anti-metastatic effect (Eckhardt et al., 2020a; Chi et al., 2024a, 2024b), while TGF-ß-Smad2/3 signaling often results in metastatic progression (Bertrand-Chapel et al., 2022). Due to previous studies demonstrating reduced GDF10 mRNA in tumors relative to healthy tissue, along with evidence that perturbed BMP signaling enhances cancer progression (Tzavlaki and Moustakas, 2020), it was hypothesized that increased GDF10 expression would reduce primary tumor growth and the incidence of metastases. While our data illustrate that GDF10 had no effect on primary tumor growth in a model of triple-negative breast cancer, there was an increase in lung metastatic burden, indicating that GDF10 may facilitate metastasis. Additionally, given recent evidence that GDF10 expression is reduced in cachectic muscle compared to healthy control (Bonetto et al., 2016; Goncalves et al., 2018; Rupert et al., 2021), and that upregulated GDF10 improved the muscle phenotype in sarcopenic mice (Kurosawa et al., 2021; Uezumi et al., 2021), we hypothesized that increased GDF10 in cachectic muscle would also improve muscle function. However, increased GDF10 expression accelerated skeletal muscle atrophy in the C-26 mouse model of cachexia.

Despite previous studies characterizing GDF10 as a potential tumor-suppressor across multiple types of cancer (Dai et al., 2004; Zhou et al., 2019; He et al., 2022), our results found no effect of GDF10 on primary tumor volume or growth when mice with breast cancer were systemically pretreated with rAAV: GDF10. This lack of effect could be attributed to the method and route of gene therapy administration. For example, studies that demonstrated GDF10-induced tumor volume reduction did so through genetically transducing the cancer cells with GDF10 *in vitro* prior to *in vivo* tumor inoculation (Dai et al., 2004; Zhou et al., 2019; He et al., 2022). In contrast, our experimental procedure involved pre-treating the mice for two weeks with a recombinant AAV that expressed GDF10 prior to inoculation with tumor cells. The latter method allows the mouse to produce the protein systemically prior to tumor cell injection, potentially enabling GDF10 to influence the tumor microenvironment. This tumor microenvironment is continuously evolving, with a complex and dynamic relationship with the primary tumor (Gerstberger et al., 2023). Pre-treating mice with rAAV: GDF10 may exert effects on cells that can influence the tumor microenvironment, in contrast to the previous studies where GDF10 activity was restricted to the primary tumor cells. Other studies have typically used heterotopic murine models, involving the subcutaneous injection of cancer cells (Dai et al., 2004; Zhou et al., 2019; He et al., 2022). In contrast, we used an orthotopic model where we injected breast cancer cells into the mammary fat pad. Whilst heterotopic models may reflect some aspects of the primary tumor microenvironment (i.e., the presence of stromal cells), the environment is likely to differ from the mammary gland (Gerstberger et al., 2023; Serrano et al., 2023). Indeed, the growth of the primary tumor is not only influenced by cellular and physical changes within the tumor cells, but also encompasses infiltrating and resident immune cells, stromal cells, blood vessels, nerves and extracellular matrix (Gerstberger et al., 2023). Thus, orthotopic models may evoke a more suitable and accurate cancer environment with which to model spontaneously arising metastasis (Cai et al., 2022), allowing for GDF10 to have an influence on the entirety of the tumor microenvironment, perhaps accounting for differences between existing literature and our findings.

While we found no GDF10-induced change in primary tumor growth in mice administered breast cancer cells, we found that systemic rAAV-mediated GDF10 administration resulted in increased metastatic burden in the lungs. These data indicate that increased systemic GDF10 expression may have facilitated one or more of the steps of metastasis, including epithelial-mesenchymal transition (EMT), migration, intravasation, circulation, extravasation, invasion and/or colonization (Gerstberger et al., 2023). While we do not know which metastatic step(s) is regulated by GDF10, the increase in lung metastatic burden was associated with increased Smad3 phosphorylation within the primary tumor. Smad3 is a key mediator of TGF-ß signal transduction and is known to promote EMT, cell invasion and metastasis (Xie et al., 2018). Other studies have observed that increased Smad3 signaling within a breast primary tumor is positively correlated to lymph node infiltration and metastases (Oueslati et al., 2023). Overall, these data indicate that increased GDF10 expression may facilitate breast cancer metastasis, in part, by increasing Smad3 signaling. Future studies could include taking histological examination of metastatic lungs to observe whether GDF10 administration increased tumor size or tumor abundance. Moreover, further research is required to elucidate which specific step(s) of the metastatic process are regulated by GDF10.

Sartori and colleagues reported previously that impaired BMP signaling in skeletal muscles contributes to the cachectic disease phenotype (Sartori et al., 2021). An analysis of muscle transcriptomic datasets from mouse models of cachexia and data from our own studies indicated reduced transcription of GDF10 in cachectic muscles. Based on these collected observations, we hypothesized that increasing GDF10 expression would conserve muscle mass in a mouse model of cancer cachexia, in part, by increasing BMP signaling. Contrary to our hypothesis, however, GDF10 significantly exacerbated cancer-induced muscle atrophy, which was associated with evidence of enhanced TGF-ß-Smad2 signaling and elevated K-48 linked polyubiquitin as a surrogate marker of protein ubiquitination and degradation. Our novel findings indicate that GDF10 may signal through the canonical TGF-ß pathway, at least in the context of cancer cachexia (Sartori et al., 2013). This finding is consistent with a recent study showing that GDF10 overexpression in C2C12 muscle cells increased Smad2/3 signaling (Kokabu et al., 2025), suggesting that increased GDF10 may cause muscle atrophy or worsen muscle health. Therefore, future studies should investigate the receptor interactions of GDF10 to determine its canonical signaling axis.

While there are no previous studies linking GDF10 to cancer cachexia, research has been conducted on the potential therapeutic effect of GDF10 in muscle wasting associated with sarcopenia, showing improvements in muscle mass and function in GDF10-treated sarcopenic mice (Kurosawa et al., 2021). While both sarcopenia and cancer cachexia are muscle wasting disorders, their pathological differences may explain the contrasting outcomes for GDF10 treatment in the sarcopenia study and our cachexia data. For example, sarcopenia is characterized by intramuscular fat infiltration that is associated with an overall increase in body fat mass (Kurosawa et al., 2021), whereas fat is often depleted in patients with cancer cachexia (Baracos et al., 2018). GDF10 is highly expressed in adipocytes and fibroadipogenic precursor (FAP) cells and is known to inhibit adipogenesis (Hino et al., 2004; Kim et al., 2025). Recently, Kim et al. (2025) found that GDF10 overexpression inhibited high fat diet-induced fat infiltration into tongue and limb muscles of mice (Kim et al., 2025). Therefore, it is plausible that the therapeutic effect of GDF10 in aged mice (Uezumi et al., 2021) could, in part, be explained by reduced fat infiltration into the muscle, rather than a direct therapeutic effect on the muscle fibers *per se*. Future experiments are required to gain more insight into why GDF10 might be beneficial in aged muscle but not in cachectic muscle.

Based on evidence in the literature, we hypothesized that GDF10 would be therapeutic in both metastatic and cachectic settings through the BMP-Smad1/5/9 signaling axis and/or suppression of TGF-ß-Smad2/3 pathways. However, in our 4T1.2 breast cancer metastasis and C-26 cancer cachexia models, we found that GDF10-induced TGF-ß-mediated-Smad3 and Smad2 phosphorylation, respectively. Our novel findings suggest that GDF10 may not signal preferentially through canonical BMP signaling. In fact, phylogenetic tree analysis has shown that GDF10 is potentially more aptly defined as an intermediate between the TGF-ß/activin and BMP/GDF ligand subgroups, thereby explaining the duality of its exerted effects (Hinck, 2012). In agreement with our data, Li et al. (2015) and Upadhyay et al. (2011) maintain that GDF10 signals through TGF-ßRI and TGF-ßRII receptors that lead to the phosphorylation Smad2/3 in neurons and mammary gland epithelial cells (Upadhyay et al., 2011; Li et al., 2015). Combined, these reports suggest that GDF10 may be an atypical BMP, whose signaling may be context- and cell-dependent. Nonetheless, our novel findings illustrate GDF10 may be a potential driver of metastatic and cachectic progression through the TGF-ß-Smad2/3 signaling axis.

In summary, our studies considered for the first time the therapeutic prospects of enhanced *in vivo* GDF10 overexpression in mouse models of metastatic breast cancer and colon cancer-associated cachexia for the first time. The intravenous administration of GDF10-expressing rAAV vectors significantly increased metastatic burden in the lungs of mice bearing 4T1.2 mammary tumors and exacerbated muscle wasting in a C-26 mouse model of cancer cachexia. In both gold-standard models for metastasis and cachexia, it was observed that GDF10 increased canonical TGF-ß signaling, thereby contradicting our initial hypothesis that GDF10 acts as a typical BMP ligand to activate Smad1/5/9 signaling. Further investigation is merited to delineate the precise role that GDF10 plays in cancer progression and its potential place in cancer therapeutics. Overall, our study highlights that inhibition of GDF10 may be a prospective therapeutic strategy to reduce breast cancer metastasis, and cachexia associated with cancer.

## Data Availability

The raw data supporting the conclusions of this article will be made available by the authors, without undue reservation.
